# Application of botanical products as nutraceutical feed additives for improving poultry health and production

**DOI:** 10.14202/vetworld.2023.369-379

**Published:** 2023-02-25

**Authors:** Karim El-Sabrout, Ayman Khalifah, Birendra Mishra

**Affiliations:** 1 Department of Poultry Production, Faculty of Agriculture (El-Shatby), Alexandria University, Alexandria, Egypt; 2 Department of Livestock Research, Arid Lands Cultivation Research Institute, City of Scientific Research and Technological Applications (SRTA-City), Borg El Arab, Egypt; 3 Department of Human Nutrition, Food and Animal Sciences, University of Hawaii at Manoa, 1955 East-West Road, Honolulu, HI, 96822, USA

**Keywords:** antioxidant, egg production, essential fatty acids, fertility rate, immunological response, meat quality, nutraceuticals, welfare

## Abstract

Poultry is one of the most consumed sources of animal protein around the world. To meet the global demands for poultry meat and eggs, it is necessary to improve their nutrition to sustain the poultry industry. However, the poultry industry faces several challenges, including feedstuff availability, the banning of antibiotics as growth promoters, and several environmental stressors. Therefore, there is a critical need to include available nutraceuticals in the diet to sustain the poultry industry. Nutraceuticals are natural chemical substances that positively influence animal physiological and productive traits. Botanical products (such as fenugreek seeds, ginger roots, and olive leaves) are among the most commonly used nutraceuticals and are gradually gaining popularity in the poultry industry due to their immense benefits in nutrition and therapeutic properties. They can be added to the diet separately or in combination (as a natural antioxidant and immunostimulant) to improve poultry health and production. Botanical products are rich in essential oils and essential fatty acids, which have multiple benefits on the animal’s digestive system, such as activating the digestive enzymes and restoring microbiota balance, enhancing poultry health, and production. These nutraceuticals have been shown to stimulate the expression of several genes related to growth, metabolism, and immunity. In addition, the essential oil supplementation in poultry diets up-regulated the expression of some crucial genes associated with nutrient transportation (such asglucose transporter-2 and sodium-glucose cotransporter-1). Previous studies have suggested that supplementation of botanical compounds increased broiler body weight and hen egg production by approximately 7% and 15%, respectively. Furthermore, the supplementation of botanical compounds enhanced the reproductive efficiency of hens and the semen quality of roosters by 13%. This review article discusses the significant effects of some botanical products in the poultry industry and how they can benefit poultry, especially in light of the ban on antibiotics as growth promoters.

## Introduction

The global poultry industries, particularly in developing countries, face feed shortages year after year. The continued use of conventional feed additives from plant or animal sources in poultry diets has become a critical issue due to the high competition among livestock species and industrial purposes. This leads to an increase in the price of these feedstuff and livestock products. There is a global trend toward finding healthier natural alternatives to synthetic pharmaceuticals and therapeutic drugs in poultry farms [1–3]. Therefore, discovering alternative and readily available feed additives is essential to protecting the poultry industry, particularly in developing countries.

The use of alternatives for feed additives in the poultry diet has recently been considered. The main purpose is that these feed additives should produce safe and high-quality food [4–6]. Nutraceuticals offer these advantages and should be considered as alternative feed additives. Nutraceuticals, also known as bioceuticals, are natural chemical compounds (natural pharmaceuticals/therapeutic) that modify and maintain normal physiological functions that support the healthy host [7]. Nutraceuticals are divided into plant essential oils [8], and established nutraceuticals, such as omega-3, 6, and 9 fatty acids [9]. They are frequently used in poultry diets as alternative feed supplements to enhance animal productivity and welfare due to their numerous growth and health benefits [2, 3, 10]. These compounds exert several biological functions in poultry and may enhance their production (obtaining high-quality products) and welfare. They also can potentially improve the balance of the intestinal microbiota, which is important in regulating metabolism, intestinal epithelial proliferation, and vitamin synthesis [10, 11]. Nutraceuticals are developed as nano-substances to treat several diseases, including nutritional deficiency (malnutrition), and coccidiosis. Furthermore, nutraceuticals in poultry diets can stimulate various gene expressions related to growth, metabolism, and immunity [12].

**Figure-1:**
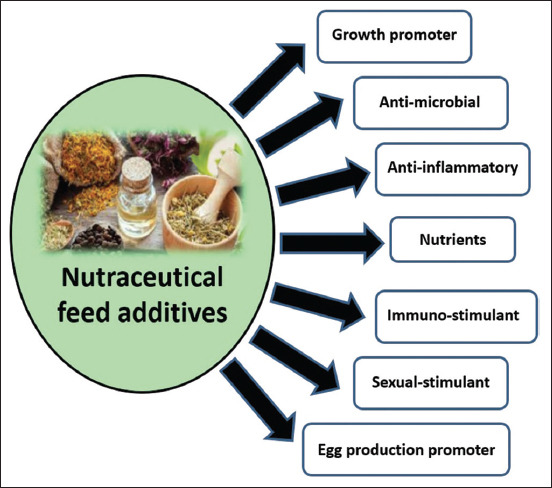
Schematic overview of the biological functions of botanical products as nutraceuticals added to poultry diets.

Botanical products (such as medicinal seeds and essential oils) are one of these nutraceuticals that are gradually gaining popularity in poultry farms due to their nutritional value and therapeutic properties (Figure-1), as well as leaving no residue in products [13]. Essential oils from plants have been routinely used in chicken farms to keep birds healthy and improve their productive performance. These essential oils include active components that positively impact physiological functions and medicinal features, such as anti-inflammatory and antibacterial effects [8, 14]. Therefore, this review discusses the impacts of using commercially available botanical products in the Middle East and beyond on poultry health and production. This article also emphasizes their properties, doses, biological functions, and welfare impact for future use in the poultry industry.

## Effect of Botanical Products on Poultry Production and Health Status

Botanical products are natural and vital substances widely used as dietary additives in the poultry industry. Their nutritional and medicinal properties positively influence performance (Table-1) [8, 15–35]. They contain phytobiotics, phenols, flavonoids, tannins, and essential oils, which have many roles in the birds. They can benefit a broad range of poultry species by improving digestion and promoting health [3, 10, 36, 37]. As a result, they perform a variety of vital roles in enhancing poultry productivity and immune functions. In addition, botanical or herbal products are considered phytogenic feed additives for poultry birds to improve egg weight and ovary characteristics and decrease yolk trimethylamine levels in laying hens [38]. Dietary supplementation of different botanical feed additives, such as camphor (50 mg/kg of feed), can enhance seminal characteristics and the reproductive performance of roosters [39].

**Table-1:** The key health and production benefits of nutraceutical/botanical product supplementation in poultry diets.

Botanical additives	Studied traits	Species	Key benefits	References
Rosemary and Cinnamon essential oils	Egg production, meat quality, blood biochemical, immunity, and antioxidative status	Laying chickens Broiler chickens	Improved hen performance, liver and kidney functions, immunity, antioxidative capacity, and egg production quality. Cinnamon oil has hypocholesterolemic and antioxidant properties, improving broiler meat quality.	[8, 16]
Pumpkin (*Cucurbita maxima*)	Egg production and quality	Laying chickens	Pumpkin can be added to the laying’s diet (10%) to improve egg production and quality (by reducing bad cholesterol levels).	[31]
Garden cress (*Lepidium sativum*)	Birds’ health and production	Broiler chickens	Garden cress can be added to the broiler’s diet (up to 1%) to improve the broiler’s body weight, biological performance, and health status.	[32, 33]
Fenugreek (*Trigonella foenum-gracum*)	Growth performance and live body weight	Broiler chickens	Fenugreek addition (1–1.5%) in the broiler diet has some growth-promoting effects on broiler chicken, improving the feed conversion ratio and body weight of birds.	[23]
	Carcass characteristics and meat quality	Broiler chickens	Better product quality was observed when fenugreek seed was added up to 3% in the commercial broiler diet.	[24]
Black cumin (*Nigella sativa*)	Egg production and quality	Laying chickens	Improve egg production, weight, eggshell thickness, and haugh unit. Reduction in yolk cholesterol content.	[25]
	Poultry health and production	Broiler and laying chickens	Promotes bords’ health and production performance. Plays a significant role as a natural antioxidant and immunostimulant.	[27]
	Productive performance, oxidative status, and meat quality	Broiler chickens	Improving broiler performance and meat quality by enhancing antioxidant activities and suppressing lipid peroxidation in meat.	[26]
Ginger (*Zingiber officinale*)	Growth performance and antioxidative status	Broiler chickens	The supplementation of ginger at the level of 5 g/kg improved the antioxidant status and bodyweight of broilers	[28]
	Feed conversion ratio, body weight, and humoral immunity	Broiler quails	Improving birds’ feed conversion ratio, body weight, and humoral immunity.	[29]
	Egg production and quality	Laying quails	Oral administration of 100–150 μL/kg body weight of ginger essential oil to quails positively affects egg weight and reduces cholesterol.	[30]
Thyme (*Thymus vulgaris* L.)	Growth, body weight, egg production, and quality	Broiler and laying chickens	Improvement in broiler’s growth and body weight. Improvement in egg production and egg nutritive value of laying hens and reduction in yolk bad cholesterol and triglyceride levels.	[15, 17-19]
Turmeric (*Curcuma longa*)	Antioxidant enzymes’ activity and body weight	Broiler chickens	Turmeric powder (0.5 g/kg diets) improved final body weight, feed conversion ratio, digestibility, and lipid status in broiler chicks under heat stress conditions.	[20, 21]
	Egg production and quality	Laying chickens	Improvement in egg production. Eggs’ cholesterol was reduced by 25% when layers were fed 4% turmeric in the diet.	[22]
Chamomile (*Matricaria* spp.)	Birds’ health and production	Broiler chickens	Adding 200–400 mg/kg of chamomile oils to broiler diets improves weight gain and final body weight and decreases microbe activities.	[34, 35]

To feed the growing human population, current poultry breeds are developed for higher production of meat and eggs. However, these breeds are metabolically more active, resulting in the production of cellular free radicals. These free radicals alter cellular functions by accelerating oxidative stress, inflammation, and immunosuppression. Recently, researchers have tested several botanical compounds and their bioactive molecules, which scavenge free radicals and restore the cellular functions required for poultry health and production.

## Thyme (*vulgaris L.*)

Thyme (*T. vulgaris* L.) is one of the most common herbal medicinal plants worldwide that belong to the *Lamiaceae* family. It is widely used in human food to provide a distinct flavor. Due to its antioxidant, antibacterial, and therapeutic characteristics, it is usually used in livestock farms (as an alternative to antibiotics) for enhancing animal productivity and health [40–42]. The essential oil in dry thyme (active extract) is a combination of monoterpenes, mainly thymol (2-isopropyl-5-methylphenol) and phenol isomer carvacrol (2-methyl-5-(propan-2-yl) phenol) [43]. Thyme also contains phenolics, some biphenylics, and flavonoids which have been shown to have antioxidative properties and other benefits for birds [41–46]. Essential oil phenolic compounds increased catalase activity, detoxifying hydrogen peroxide and converting lipid hydroperoxides to nontoxic substances [47]. Thyme extracts are advised in laying farms to improve egg quality, especially the fatty acid profile in the yolk [48]. Lipid profiles of the egg can be modified by feeding hens plant seed oils to reduce the content of n-6 fatty acids while increasing the content of n-3 fatty acids [49, 50]. Layer’s diet enriched with dry thyme leaves at 2% has several positive impacts on laying hens’ physiological and productive performance and their antioxidant activity [15]. Thyme leaves significantly influence egg production quality and nutritional value by decreasing bad yolk cholesterol (low-density lipoprotein [LDL]) and total saturated fatty acid concentrations and increasing omega-3 fatty acid concentrations. It also raises the a-linolenic acid level of eggs while significantly reducing palmitic acid content in the yolk. Furthermore, dietary thyme at 2% can reduce yolk malondialdehyde, unhealthy blood cholesterol, and triglyceride levels. Blood bad cholesterols (such as LDL) reduction may be related to 3-hydroxy-3-methylglutaryl- coenzyme A (HMG-CoA) reductase activity inhibition, which is one of the enzymes involved in regulating cholesterol metabolism; thus, cholesterol synthesis reduction [16]. The previous studies [15, 17, 18, 48, 51] reported that dietary thyme supplementation could enhance poultry growth performance and egg production and the humoral immune response without harming the birds. In addition, dietary supplementation of thyme (1–5 g/kg) in broiler diets as a growth promoter increased feed intake, feed conversion efficiency, and final body weight. It can also increase birds’ meat dressing percentage and quality [17, 19, 52]. Thyme extracts also have the potential to improve broiler meat oxidative stability by interrupting free radicalchains, resulting in a product with higher oxidative stability [53]. Its mode of action is related to intramuscular fat stimulation and flavor amino deposition. Phytogenic products containing thymol and carvacrol enhanced broilers’ growth performance, digestive enzyme activities, antioxidant enzyme activities, and retarded lipid oxidation [54]. In the poultry diet, phytogenic feed additives functioned for overall broiler gut antioxidant capacity. A 5% reduction in dietary energy and protein intake primed important antioxidant responses, particularly on phytogenic addition [55]. Diet-type interactions with phytogenic feed additives were determined for critical antioxidant and cytoprotective genes (such as nuclear factor erythroid 2-like 2 pathway) and the total antioxidant capacity in the proximal gut. The overall antioxidant response along the broiler gut was increased by reduced dietary energy and protein intake, and the addition of phytogenic feed additives consistently increased it. On the other hand, thyme contains flavonoids, which can increase the activity of Vitamin C, which acts as an antioxidant and thus improves immune function [54, 56]. It also plays an important role in reducing stress on birds, especially during the hot seasons (such as summer), and increasing feed intake, metabolism, and body weight in broiler chickens [57]. Thyme essential oils can improve birds’ productivity by increasing the secretion of digestive enzymes and improving nutrient utilization through improved liver function [19, 58, 59]. Thyme’s antibacterial activity was associated with increased broiler productivity [40]. Broilers fed a diet supplemented with thyme had higher productivity, which they attributed to increasing apparent fecal and crude protein digestibility [60]. It has been reported that essential oils (25% thymol and 25% carvacrol) supplementation on growth performance, gut lesions, intestinal morphology, and immune responses of broiler chickens infected with *Clostridium perfringens* [45]. The supplementation induced gut lesions and increased crypt depth in the ileum. It also downregulated the claudin-1 and occludin mRNA expression, up-regulated the mRNA expression of interleukin-1β, and tended to increase toll-like receptor 2 mRNA expression in the ileum, and improved mucosal secretory immunoglobulin A production. These studies concluded that dietary essential oil supplementation alleviated gut lesions and improved the ratio of villus height to crypt depth in the treated birds. Furthermore, they revealed that essential oils supplementation (120 and 240 mg/kg) increased the serum antibody titers against the Newcastle disease virus and improved birds’ immune responses. Essential oil supplementation quadratically and linearly up-regulated the expression levels of critical genes associated with nutrient transportation (such as glucose transporter-2 [GLUT2], sodium-glucose cotransporter-1 [SGLT1], and Sodium-coupled neutral amino acid transporter 1), and barrier function of tight junction protein-1 [61]. These findings indicate that essential oil positively affects growth, immunity, and intestinal health in broilers, and 200 mg/kg of essential oil is recommended in the broiler diet.

## Turmeric (*Curcuma longa L.*)

Turmeric (*C. longa* L.), a member of the *Zingiberaceae* family, is a rhizomatous herbaceous plant belonging to the ginger family. Curcumin is a major component of turmeric and is commonly used as a spice and food-coloring agent [62]. It has been considered a natural polyphenol nutraceutical substance and is well known for reducing oxidative stress and repairing the damage caused by oxidative stress. According to Sharma *et al*. [63], curcumin has been identified for various biological activities, including antioxidant and anti-cancer effects. Furthermore, several studies have shown that curcumin has anti-inflammatory properties and lipid and cholesterol-reducing effects [64, 65]. It can also be used in animal diets, such as broiler diets, to mitigate heat stress [20, 21]. Furthermore, it can reduce free radicals; reactive oxygen species (ROS), and reactive nitrogen species (RNS), by activating glutathione, catalase, and superoxide dismutase, and limiting ROS-producing enzymes [66, 67]. Thus, it becomes a vital medical and antioxidant substance in chicken feeds, especially in tropical areas where high temperatures throughout the year can delay growth, decrease egg production, and increase disease outbreaks and mortality of birds [68]. These issues worsen when the birds are subjected to heat stress [69, 70]. Sadeghi and Moghaddam [20] observed that adding turmeric (0.5%) to broiler diets during heat stress raised blood concentrations of triiodothyronine (T_3_) and thyroxine (T_4_), feed consumption, body weight, and decreased bird feed conversion ratio. Moreover, El-Maaty *et al*. [71] revealed that the addition of turmeric to broiler diets (0.5%) increased final body weight (11%), feed conversion ratio (17%), crude protein digestibility (5%), and high-density lipoprotein (HDL) (28%) while lowering creatinine, triglycerides, cholesterol (>15%), LDL (39%), and very-LDL (VLDL) (>13%) during thermal stress conditions. Triglycerides are secreted from the liver into the blood by triglyceride-rich lipoproteins; thus, reduced hepatic lipogenesis lowers triglyceride concentrations in plasma [72]. The reduction of bad cholesterols (LDL and VLDL) may be related to the inhibition of HMG-CoA reductase (one of the enzymes involved in regulating cholesterol metabolism in animals) activity [16].

Turmeric and other natural plant extracts can be added to poultry diets in small amounts to enhance several productive and health benefits [22, 73]. Supplementing turmeric in the poultry diet at a 1%–5% concentration improves feed intake and significantly lowers cholesterol in poultry products. In laying hens, dietary supplementation of turmeric at 1% and 4% reduced eggs’ cholesterol by 16% and 25%, respectively [22]. Compared to a control group, birds fed a high carbohydrate content supplemented with turmeric for a month before sexual maturity produced higher eggs (20%). The active turmeric extract (curcumin) influences lipid metabolism and inhibits peroxidation [74]. It stimulates bile production, which is necessary for lipid emulsification [75]. Turmeric supplementation can improve liver function and vitellogenin production [22]. Turmeric enhances liver function by reducing serum levels of glutamic pyruvic transaminase/alanine aminotransferase and glutamic oxaloacetic transaminase/aspartate aminotransferase [22, 76].

## Fenugreek (*Trigonella foenum-graecum*)

Fenugreek seeds (*T. foenum-graecum*) are known for their medicinal properties, including antibacterial and anti-inflammatory effects [77, 78]. They are also high in protein, fat, total carbohydrates, minerals, biotin, and trimethylamine, stimulating the animal’s appetite [77, 79]. Galactomannan is the main polysaccharide in fenugreek seeds, representing approximately 50% of the seed weight [80]. It has been recognized as an anti-diabetic nutraceutical due to its capacity to decrease blood glucose levels. Adding fenugreek seed powder (1%–1.5%) to the broiler diet improved the feed conversion ratio and increased final body weight in birds [23]. While adding up to 3% fenugreek in broiler diets can improve birds’ body weight gain and carcass quality [24]. Adding 1% fenugreek to laying hens’ diet improved feed intake and egg yolk color, particularly in the second production cycle [81]. Fenugreek supplementation at 0.4% in laying diets improved egg production and quality [82]. These studies have suggested that supplementation of fenugreek can potentially improve meat and egg production and their quality.

## Black Cumin (*Nigella sativa*)

Black cumin seeds (*N. sativa*) are well known for their medicinal and therapeutic characteristics. They contain antioxidants, volatile oils, alkaloids, and several pharmacologically active substances, such as thymols [25, 83–85]. Dietary supplementation of black cumin enhanced broilers’ production and meat quality by increasing antioxidant activities and inhibiting meat lipid peroxidation [26]. Black cumin seeds are also rich in essential oils and essential fatty acids, and have multiple benefits on the animal’s digestive system, such as activating the digestive enzymes, restoring microbiota balance, and increasing nutrient absorption [86, 87], and this may be one of the reasons behind birds’ production and health improvements. Azeem *et al*. [27] found that black cumin enhances poultry productivity and immunological resistance. They reported that black cumin seeds play bioactive roles in the avian body as a natural antioxidant and immunostimulant. Black cumin seeds added to the chicken diet at the dose rate of 10,000–30,000 mg/kg increased nutrient utilization, body weight gain, immunological response, and lower fatty acid levels in the products [88]. Furthermore, *N. sativa* meal could be added up to 15% in growing quail diets with no negative effects on productive performance. Compared to the control, the inclusion of 10% *N. sativa* meal in the quail diet resulted in the best values of live body weight and daily weight gain [89]. The up-regulated expression of some nutrient transporters, such as SGLT1 and GLUT2, may be another reason for birds’ production and health improvements [61].

## Olive (*Olea europaea L.*)

Olive by-products (leaves, cake or pulp meal, and oil) are among the most important nutraceutical substances because of their nutritional and healthful effects. Polyunsaturated fatty acids (PUFA), polyphenols, and minor phytochemical compounds in olive products improved productivity and the immune system by influencing metabolism processes and the synthesis of white blood cells and cytokine [45, 90–93]. Olive leaves, cake meal (a by-product of olive oil extraction), and oil are used in poultry diets as a source of energy and antioxidant compounds [94]. Olive leaves have high antioxidant activity, originating from phenolics, thus exhibiting strong preventive effects against oxidation [95, 96]. Olive cake meal has a high nutritional value (crude fat, 13%–18%; crude proteins, 9%–10%) [97, 98]. It is rich in lignin and includes a high content of non-starch polysaccharides [94, 99]. Furthermore, it contains Vitamin E, calcium, iron, potassium, magnesium, sodium, and phosphorus [100]. Olive cake meals are found worldwide and used as a plant source for broiler diets at a fair price in several countries [101]. Olive oil is distinguished from other vegetable oils by its high oleic acid content (the most common monounsaturated fatty acid in the diet) [102]. The importance of olive oil stems from its high levels of monounsaturated fatty acids and the presence of low-represented components such as alpha-tocopherol, phenolics, chlorophyll, and carotenoids [103]. Adding 5% olive oil to broiler diets can decrease serum triglyceride levels while increasing HDL-cholesterol levels in treated birds [104]. This addition reduced saturated fatty acid contents in breast and drumstick meat and increased the total unsaturated fatty acid contents. Moreover, supplementing 2% or 5% olive oil to layer diets boosted yolk color, increased total yolk unsaturated fatty acids, and decreased serum cholesterol concentration in laying hens [102]. Laying hens’ diet supplemented with dried olive pulp at rates of 5% and 6% improved egg nutrition quality by increasing the PUFA percentage in eggs, decreasing that of saturated fatty acids, and improving the PUFA to saturated fatty acids ratio [105]. Olive by-products beneficial effects could be due to their high content of vital and biological compounds, such as monounsaturated fatty acids, PUFA, and polyphenols, as well as their high antioxidant capacity [106]. Furthermore, olive oil improves the absorption of Vitamin A, another fat-soluble vitamin, which might increase HDL-cholesterol levels in the sera of laying hens [107]. Olive oil supplementation also increased calcium concentration in non-ruminant animal livers, such as poultry, and made it more available for absorption [108]. Hens can absorb calcium through their digestive systems and mobilize calcium from the medullar bones [109]. On the other hand, olive oil is a good solvent of Vitamin D and thus can improve calcium concentrations in eggshells [110]. These studies demonstrated that supplementing olive products and by-products has beneficial effects on poultry meat and egg production.

## Ginger (*Zingiber officinale*)

Ginger roots (*Z. officinale*) are rich in volatile oils, gingerols, and zingerone and are widely used as medicinal plants worldwide [111]. Ginger roots enhance digestive enzymes and antioxidative activities in birds [112]. Dietary supplementation of 5000 mg/kg ginger powder as a nutritional addition in broiler diets increased antioxidant capacity and serum metabolites [28]. Quails fed a ginger-supplemented diet (125 mg/kg) had the best feed conversion ratio, body weight, and humoral immunity [29]. Furthermore, the addition of ginger aided in the optimization of the lipid profile in blood serum and enhances the bird’s antioxidative status. Bee propolis (500 mg/kg) mixed with ginger powder (125 mg/kg) displayed better growth performance and poultry health [29, 113]. Dietary supplementation of ginger powder (10–15 g/kg) in laying diets improved the laying performance and serum antioxidant status of hens [114]. In addition, oral administration of 100–150 μL/kg body weight of ginger essential oil to laying Japanese quails positively influenced egg weight and decreased egg cholesterol [30]. On the other hand, adding 2.5 and 5 g/kg ginger to the broiler breeder’s diet increased the reproductive performance of males, including semen ejaculate volume and sperm concentration per ejaculate, live sperms, and viability [115].

## Pumpkin (*Cucurbita maxima*) and Garden Cress (*Lepidium sativum*)

Pumpkin (*C. maxima*) and garden cress (*L. sativum*) seeds are important medicinal plant seeds that have recently gained attention on a global scale due to their nutritional and pharmaceutical properties, leading to the development of innovative sources of feed additives. They are rich in protein (25%–30%) and an almost equal amount of healthy fats. They also have a good amount of vitamins and minerals, essential for physiological and biological functions in poultry [116–122]. Moreover, they contain phenolic components (phytochemicals) that are responsible for their high antioxidant capacity [116–124]. Pumpkin and garden cress seeds are also rich in polyphenols, flavonoids, antioxidants, and PUFA [125]. These compounds have various biological activities in bird’s body, including growth gene stimulation, metabolic process promotion, and final product quality improvement by increasing PUFA and decreasing bad cholesterol levels [12, 45, 126, 127].

Pumpkin and garden cress seeds can be added combined or separately to poultry diets to improve birds’ development and production, as well as the health and immune response [13, 116, 122, 128–130]. They contain essential aromatic oils with various nutritional and medicinal properties for birds. They are excellent sources of unsaturated fatty acids, such as linoleic acid, and saturated fatty acids, such as arachidic acid [131, 132]. Furthermore, they contain a-linolenic acid, which can be transformed into eicosapentaenoic acid and docosahexaenoic acid in the body [133].

Pumpkin seed oil is high in Vitamin E and β-carotene, which have powerful antioxidant and anti-cancer properties [120, 121, 123, 134]. It is also rich in omega-3 and omega-6 fatty acids, L-tryptophan, and some trace minerals such as zinc [135, 136]. It is recommended that laying hen diets contain 10% pumpkin to improve ether extract and healthy fatty acid levels while decreasing harmful cholesterol and fatty acids [31]. Moreover, pumpkin is a good source of crude protein and can potentially substitute soybean meal in broiler diets up to 20% without causing any adverse effect on the bird’s internal organs [122].

Garden cress oil contains tocopherol (a natural antioxidant), carotenoid, oleic acid, and a-linolenic acid, which reduce various types of radicals [131, 137]. Garden cress can be added, as a potential feed additive, to the broiler’s diet at the level of 0.75% for better biological performance and health status [32]. In addition, garden cress supplementation (1%) increased broiler feed consumption, body weight, blood globulin, behavior, and economic efficiency [33].

## Azolla (*Azolla pinnata*)

Azolla (*A. pinnata*) is a small aquatic fern plant that lives afloat on the water’s surface and can resist hot temperatures. It provides a carbon source and a favorable environment for algae growth and development. It is rich in proteins (25%–35%), minerals (10%–15%), and vitamins (20%–30%) and has a low fiber content (11%–13%) [138–141]. Furthermore, it contains a good amount of carotenoids, which play a useful biological role that contributes to therapeutic effects, such as immunomodulators, antibacterial, and anti-inflammatory effects, and improves poultry productive performance and product quality [142]. However, due to its high nutrient content, Azolla is used as a feedstuff in poultry and livestock diets. It can be used as a low-cost animal feed and a partial replacement for high-priced traditional proteins in poultry diets. According to Mishra *et al*. [138] and Azolla can be used in poultry diets at 25%–30% for chickens and a higher rate of 40%–45% for ducks and geese [138, 141]. The high enzyme concentration in the *Azolla* plant may be due to active components in this plant, which can inhibit free radical activity within the animal’s body as one of the most important natural antioxidants [138].

## Chamomile (*Matricaria spp.*)

Chamomile flower (*Matricaria* spp.) is one of the most common herbs used for medicinal purposes worldwide. Egyptian chamomile (*Matricaria recutita*) is famous for its high-quality; therefore, large quantities of this plant are exported to European countries. It contains flavones (such as apigenin), coumarins, and essential volatile oils (1%–2%) (such as bisabolol oxide A and B (25%–50%), and chamazulene (10%) [143]. Besides its medicinal properties, it has antioxidant, anti-inflammatory, antimicrobial, and cholesterol-lowering activities [143–147].

It was reported that feed conversion, weight gain, and market body weight were significantly improved with 200–400 mg chamomile oils for each kilogram broiler diet [34]. Chamomile also has a beneficial effect on broiler plasma cholesterol and glucose. It was found that increasing doses of chamomile in the feeding ration decreased the numbers of coliform microbes in the digestive tract of chicks and reduced the population of *Clostridium perfringens* [35]. In laying Japanese quails, supplementing chamomile at 0.50 g/kg of the diet improved productivity, reproductive performance, and economic efficiency [144]. Furthermore, including 2.5–5 g chamomile/kg in a Japanese quail diet reduces the behavior of aggressive pecking and improves birds’ welfare [147].

## Conclusion

Nutraceuticals are bioactive chemical substances found in several botanical products. They displayed promises as effective feed additives for sustaining poultry productivity while improving their health. Botanical products have been widely used as dietary supplementation in poultry diets due to their nutritional and therapeutic properties. Therefore, botanical products are recommended as natural feed additives and alternatives to any artificial chemical material. These natural substances can be added to the poultry diet individually or in combination to improve the physiological, productive, reproductive, and immunological parameters which impact poultry welfare and their quantitative and qualitative productivity. This review summarized the botanical products’ health and production benefits as nutraceuticals. These nutraceuticals can potentially be used in broiler and layer farms as natural growth and egg production promoters and aid in the sustainability of the poultry.

## Authors’ Contributions

KE: Conceptualization, supervision, visualization, and writing–original draft. KE and BM: Data curation and writing–review and editing. KE, BM, and AK: Formal analysis, investigation, and resources. All authors have read, reviewed, and approved the final manuscript.
